# Association of Pregnancy Complications with Endometrial or Ovarian or Breast Cancer: A Case Control Study

**DOI:** 10.3390/medicina61010001

**Published:** 2024-12-24

**Authors:** Lin Cheng Han, Henry W. C. Leung, Heng-Jun Lin, John Hang Leung, Agnes L. F. Chan

**Affiliations:** 1 Department of Obstetrics and Gynecology, Wan Fang Hospital, Taipei Medical University, Taipei 116, Taiwan; 111150@w.tmu.edu.tw; 2 Department of Radiation Oncology, An Nan Hospital, China Medical University, Tainan 709, Taiwan; 070506@tool.caaumed.org.tw; 3 Department of Management Office for Health Data, Clinical Trial Research Center, China Medical University Hospital, Taichung 404, Taiwan; 4 Department of Obstetrics and Gynecology, Ditmanson Medical Foundation Chia-Yi Christian Hospital, Chiayi 600, Taiwan; 5 Department of Pharmacy, Kaohsiung Show Chwan Memorial Hospital, Kaohsiung 821, Taiwan

**Keywords:** preeclampsia, GDM, IUGR, endometrial cancer, ovarian cancer

## Abstract

*Background and Objectives*: The relationship between pregnancy complications and the risk of gynecological and breast cancer remains inconclusive, with limited research available. This study aimed to determine whether pregnancy complications, including preeclampsia, gestational diabetes mellitus (GDM), large for gestational age (LGA), or intrauterine growth restriction (IUGR) are associated with the development of endometrial cancer (EC), ovarian cancer (OC), or breast cancer (BC). *Materials and Methods*: This was a population-based case–control study linked to the National Health Insurance Research Database from 2008 to 2020, using ICD codes to identify parous gynecological cases (n = 6714). The propensity score matching method was used to match control groups (n = 1,153,346). Multivariable logistic regression models were used to determine the association between EC, OC, BC risk and pregnancy complications. *Results*: In adjusted multivariable logistic regression models, women with a history of preeclampsia did not have a significantly increased risk of endometrial, ovarian, or breast cancer compared to controls. Although women with GDM complications had a significantly increased risk of breast cancer, the increased risk of EC or OC was not significant. The risk of BC in women with a history of IUGR or LGA was not significant, whereas risk statistics for EC or OC in women with a history of IUGR or LGA could not be shown because of the small sample size. *Conclusions*: GDM is associated with BC risk. Future studies should aim to determine whether there is a causal relationship. Therefore, cancer screening is warranted in women with GDM.

## 1. Introduction

In 2022, ovarian cancer, endometrial cancer, and breast cancer were the most common cancers among women worldwide and in Taiwan [1,2]. The most common and established risk factors for ovarian and endometrial cancer include elevated levels of unopposed estrogen, early menarche, late menopause [3,4], grand multiparity, family history, BRCA gene mutations, advancing age, endometriosis and lifestyle factors such as obesity and diet [5,6,7,8].

Pregnancy-related factors associated with a lower risk of breast cancer include earlier age at first full-term pregnancy, increased number of deliveries, history of preeclampsia and longer duration of breastfeeding [7,9,10]. Conversely, potential pregnancy-related factors associated with higher breast cancer risk include nulliparity, older age at first pregnancy, increased exposure and/or high levels of ovarian hormones, factors associated with post-pregnancy mammary duct regression, negative estrogen (ER)- and progesterone (PR)-receptor status and a higher rate of HER2 overexpression [10]. Maternal obesity can have long-term adverse health effects, increasing the risk of breast cancer and mortality in offspring [11].

Evidence for the increased risk of endometrial cancer or ovarian cancer associated with pregnancy complications of preeclampsia and gestational diabetes mellitus (GDM) is also inconsistent. Several studies have reported that preeclampsia increases the risk of endometrial cancer or ovarian cancer [12,13,14]. Conversely, other recently published studies suggest that preeclampsia is not associated with an increased risk of endometrial or ovarian cancer [15]. Recent studies have shown that women with GDM are at an increased risk of endometrial or ovarian cancer [16,17], but other studies have reported no association between GDM and endometrial or ovarian cancer in women later in life [18,19]. This recent evidence is still inconclusive.

There are several potential mechanisms for reducing the risk of gynecological cancer, such as the protective effect of multiparous pregnancy, breastfeeding, and lack of ovulation during pregnancy, which have been reported to be related to the shift in hormonal balance (increased progesterone levels and reduced estrogen levels) [20,21]. Previous studies have reported that hormonal changes may influence malignancy, leading to estrogen-induced DNA damage and carcinogenesis [22,23]. Increased progesterone concentration during pregnancy can inhibit estrogen-driven endometrial cell proliferation and promote endometrial cell differentiation and apoptosis [24,25]. However, the protective role of progesterone in ovarian cancer was re-evaluated in a recently published study. They proposed that the transcriptional activity of the nuclear progesterone receptor (nPR) may be ligand-dependent or ligand-independent and fully integrated with other ubiquitous cell signaling pathways that are frequently altered in cancer [26]. Furthermore, no differences in circulating estradiol and progesterone levels have been found between cancer subtypes in premenopausal and postmenopausal women, and parity does not affect sex hormone levels regardless of menopausal status [27]. Another hypothesized mechanism suggested that the placenta also produces large amounts of estrogen and progesterone during pregnancy [28].

The protection of multiparous pregnancy for breast cancer, several hypotheses have been proposed to explain the biological basis of these observations, including direct effects of pregnancy hormones on breast cells and/or the effects of persistent epigenetic changes on subsequent hormone concentrations or breast tissue [29,30], but no human data fully resolve any hypothesis.

Pregnant women with preeclampsia are at risk of intrauterine growth restriction (IUGR), but no increased risk of endometrial or ovarian cancer has been observed in women with a history of preeclampsia or IUGR [15]. The underlying mechanism may involve abnormal serum concentrations of estrogen and progesterone [31,32]. The imbalance between estrogen and progesterone could also impair fetal maturation and growth in infants with IUGR [33].

The risk of endometrial cancer (EC) and delivery of large for gestational age (LGA) neonates has not been well studied, but a recently published study by Liu et al. reported that LGA delivery was associated with an increased risk of EC but not ovarian cancer (OC). This evidence further emphasizes that the protective effect of pregnancy against endometrial cancer in multiparous women is complex and multifactorial.

Because the evidence is inconsistent and controversial, we aimed to determine whether pregnancy complications, including preeclampsia, gestational diabetes mellitus (GDM), large for gestational age (LGA) or intrauterine growth restriction (IUGR), are associated with endometrial cancer (EC), ovarian cancer (OC) or breast cancer (BC).

## 2. Methods

### Data Source

We performed a case–control study from 1 January 2008 to 31 December 2020, using data retrieved from the catastrophic illness patient dataset (HV) of the Taiwan National Health Insurance Research Database (NHIRD). The HV dataset is a subset of the database of the NHIRD. Cancers are defined by the government as catastrophic illnesses. All patients with cancer or other catastrophic illnesses or injuries are issued a catastrophic illness certificate (CIC), and these patients are exempted from copayment to the National Health Insurance (NHI) plan, which covers approximately 99% of the 23 million Taiwanese residents if they receive care for their catastrophic illness. The NHIRD contains comprehensive information on healthcare utilization among Taiwan’s National Health Insurance beneficiaries, including de-identified and encrypted information on demographics, clinical diagnoses, procedures, and prescription refill records from outpatient, inpatient, and acute care settings. The disease codes in the database adhere to the International Classification of Diseases, Ninth, and Tenth Revision. All methods complied with relevant guidelines and regulations.

## 3. Study Cohort

**Figure 1:**
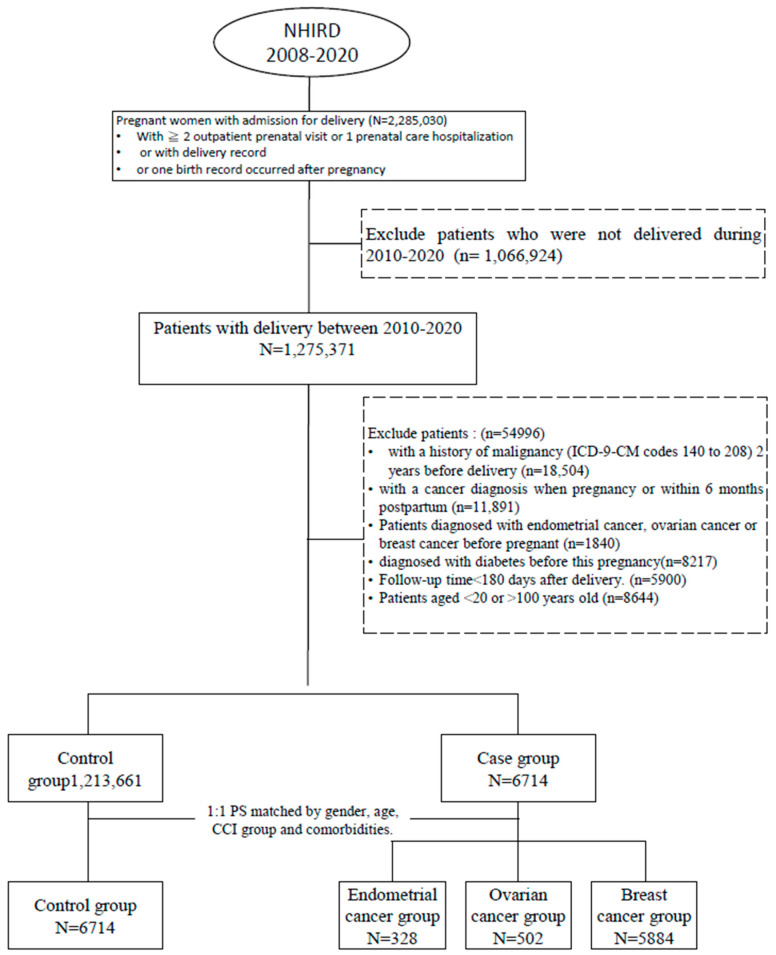
Flow chart of study population.

A total of 2,285,030 pregnant women admitted for delivery were found in the NHIRD between 1 January 2008 and 31 December 2020 (Figure 1). Patients were traced back 2 years before delivery to identify a history of malignancy or pregnancy complications, including preeclampsia, gestational diabetes mellitus (GDM), large for gestational age (LGA) or intrauterine growth restriction (IUGR), and to assess baseline comorbidity variables associated with complications and malignancy. The patient cohort was then linked to the Catastrophic Patient Registry (HV) using encrypted patient identification numbers to identify newly diagnosed primary gynecological cancers based on the International Classification of Diseases, 9th Revision, Clinical Modification (ICD-9-CM) code or the International Classification of Diseases, 10th Revision, Clinical Modification (ICD-10-CM) code for endometrial cancer (EC)(ICD-9:179; 181, 182–182.1, 182.8; 233.2, 236.0, 236.1, 621.33; ICD-10-CM: C54.1, C55, D07.0, N85.02), ovarian cancer (OC)(ICD-9:183.0–183.9; 236.2; ICD-10: C56, C56.1, C56.2, C56.9) and breast cancer (BC) (ICD-10: C50.011–012, C50.111–112, C50.611–612, C50.811–812, C50.911–912, D05.01–D05.02, D05.11–12, D05.81–82) (Supplementary Table S1).

Inclusion criteria were (1) women admitted for delivery between 1 January 2010 and 31 December 2020, to avoid preexisting malignancies and ensure adequate follow-up time; (2) patients diagnosed with relevant complications and who had received at least ≥2 outpatient prenatal visits; (3) inpatient prenatal care; and (4) patients with a birth record or a birth record that occurred after pregnancy. Patients meeting any of the following criteria in the year preceding the cohort entry date were excluded: (1) women not admitted for delivery between 1 January 2010 and 31 December 2020; (2) women with a history of malignancy (ICD-9-CM codes 140 to 208) 2 years before delivery; (3) women with a cancer diagnosis when pregnant or within 6 months postpartum; (4) women diagnosed with endometrial cancer, ovarian cancer or breast cancer before pregnancy; (5) a follow-up time < 180 days after delivery; and (6) patients aged <20 or >100 years.

## 4. Case Identification and Control Selection

We identified patients diagnosed with endometrial, ovarian or breast cancer as the case group and defined the latest diagnosis date for the three female cancers of interest as the index date. Patients without endometrial, ovarian or breast cancer were included in the control group.

## 5. Confounding Variables

Confounding variables in this study included age and comorbidities (myocardial infarction, congestive heart failure, peripheral vascular disease, hypertension, cerebrovascular disease, chronic pulmonary disease, ovarian dysfunction, female infertility, obesity, alcohol-related disease, Charlson Comorbidity Index (CCI), parity number and abortion number). CCI is a valid and reliable measure of comorbidity and can be used in diverse populations as a prognostic indicator to predict mortality in longitudinal studies. We used the Charlson Comorbidity Index to classify patient comorbidities retrieved from the NHIRD based on International Classification of Diseases (ICD) diagnosis codes. We assigned a relative weight (from 1 to 6) to each comorbidity category based on the adjusted risk of death or resource use and derived a patient’s single comorbidity score by the sum of all weights. Only comorbidities that were diagnosed at least twice during outpatient or hospital visits were retrieved from the database. Therefore, the retrieved patients with comorbidities were classified into three categories. A score of zero indicated no comorbidities. The higher the score, the more likely it was that the prediction would result in death or higher resource usage. All confounding variables are listed in Table 1.

Propensity score matching (PSM) is a statistical matching technique that attempts to assess the effect of a treatment or other intervention by elucidating the confounding factors that predict treatment receipt. It also tries to balance confounding factors between treatment groups so that they are comparable, and then we can use the observational data to derive the causal effect of treatment on the outcome [34]. PSM is calculated from the results of a logistic regression model, in which treatment exposure status was regressed on one-to-one matching of observed confounding variables using a greedy algorithm, called the 5 → 1 number matching improved macro, which was performed in hierarchical order. The “best” match is made first and the “next best” matches next, until no more matches can be made. The best matching was defined as the highest digit match on the propensity score. The algorithm proceeds sequentially to the lowest numeric match of the propensity score (1 digit: 0.1) [35].

## 6. Statistical Analysis

The chi-square test was used to compare female characteristics as categorical variables between cases and controls, and the two-way analysis of variance (ANOVA) test was used to compare groups stratified by normally distributed continuous variables. A conditional logistic regression model was used to estimate the risk of endometrial, ovarian, and breast cancers. Multivariate logistic regression analysis was adjusted for age, CCI and comorbidities. All statistical analyses were performed using the SAS software (version 9.4; SAS Institute, Inc., Cary, NC, USA) and R studio (3.5.2). Differences were considered statistically significant at *p* < 0.05.

## 7. Results

### Study Populations

After specifying the exclusion criteria, 1,275,371 pregnant women who were admitted for delivery were found in the NHIRD between 1 January 2000 and 31 December 2020 (Figure 1). After 1:1 matching, 6714 pregnant women diagnosed with cancer of interest for this study were included in the case group, and pregnant women without diagnosed cancer were included in the control group.

Table 1 presents the general characteristics of the study participants. Women with a history of EC, OC and BC had a significantly higher age at diagnosis and median age at first delivery than controls. With the exception of ovarian dysfunction, infertility, obesity and connective tissue diseases, there were no significant differences in most comorbidities between the case and control groups.

## 8. Pregnancy Complications Associated with Gynecological Cancers and Breast Cancer

Table 2 shows the relationship between the risk of EC, OC or BC and a history of GDM, IUGR, LGA and preeclampsia. In the unadjusted analysis, we observed a nonsignificant decrease in the risk of EC and BC but an increased risk of OC (OR = 1.56; 95% CI (0.73, 3.37); *p* = 0.254) in women with a history of preeclampsia compared with controls. Interestingly, women with a history of GDM had a significant and nonsignificant increased risk of breast cancer (OR = 1.13; 95% CI = 1.03–1.23; *p* = 0.010) and OC (OR = 1.01; 95% CI = 0.73–1.41; *p* = 0.933).

In the multivariable logistic analysis, we adjusted for age, CCI group and comorbidities, including hyperlipidemia, obesity, hypertension, diabetes mellitus, ovarian dysfunction and female infertility. Women with GDM complications in the case group had a significantly increased risk of breast cancer compared to those in the control group (aOR = 1.12; 95% CI = 1.02–1.23, *p* = 0.013). However, the risks of breast cancer and EC were nonsignificantly reduced, whereas no significant increase in the risk of OC was observed in women with preeclampsia.

Women with a history of IUGR and LGA had an increased (OR = 1.42, 95%I = 0.57–3.55) and decreased (OR = 0.88; 95% CI = 0.43–1.80) risk of breast cancer, respectively, but the risk was not statistically significant. Regarding the risk of EC or OC in women with a history of IUGR or LGA, statistical analysis cannot be performed due to the small sample size, and therefore, statistical risk information cannot be shown. This finding may be attributed to the continuous decline in Taiwan’s birth rate, resulting in a lower incidence of IUGR or LGA [36].

## 9. Discussion

Of the four pregnancy complications tested in the current study, only preeclampsia and GDM showed a causal relationship with the risk of developing EC, OC or BC. In the present study, preeclampsia showed no significant reduction in EC and BC risk, which is consistent with recently published reports on EC [14,19] and BC [7,13] risk outcomes. In contrast, the risk of OC increased, but not significantly, in women with preeclampsia. This result is also consistent with previously published research [37]. In summary, while previous studies have not yet reached consistent conclusions regarding the relationship between preeclampsia and BC risk, our study provides updated data from a recently published meta-analysis reporting a significant reduction in the risk of preeclampsia and BC later in life (pooled RR = 0.93, 95% confidence interval = 0.83–0.93) [30]. Therefore, we can confirm that our results are consistent with those of previous studies that preeclampsia may have a protective effect against breast cancer.

Likewise, the risk of EC or OC showed no significant association with GDM in the present study, a result supported by several studies [18,19,38]. Meanwhile, a significantly increased risk of BC associated with GDM was observed in this study, which is also consistent with several recently published studies [34,39,40]. Interestingly, similar reports were found in two previously published and recently updated studies, where they explained that Chinese Asians are less insulin-resistant than other Asian ethnic groups [41,42,43]. The reason may be due to unique biological factors that contribute to ethnic differences in adiponectin levels, inflammation and insulin resistance [43,44]. Insulin resistance is also associated with metabolic syndrome, obesity [41,42] and central obesity [41], all of which are associated with a higher risk of breast cancer. Given the rapidly increasing incidence of obesity and GDM in Asian populations, we believe we have achieved consistent results and suggest that pregnancy complications of GDM may influence the risk of BC; however, systemic and chronic inflammation may be potential mechanisms by which GDM affects BC development [41].

However, the mechanisms underlying this phenomenon remain unknown. Some studies have shown an inverse correlation with parity. The incidence of endometrial and ovarian cancers may be explained by hormonal changes that occur during pregnancy. However, current evidence suggests that the fetal antigen hypothesis and the extracellular vesicle hypothesis may be potential mechanisms for the protective effect of increased parity on cancer development [5]. The fetal antigen hypothesis postulates that certain proteins are released into the maternal circulation via placental/fetal cells during pregnancy. These fetal antigens are thought to stimulate the maternal immune system and produce long-lasting natural immunity. When these mothers fight tumors that produce the same fetal antigens, they produce long-lasting immune responses to proteins expressed by endometrial cancer cells [5,42]. Placental extracellular vehicles (EVs) may deliver the maternal immune response to the fetal tissue. Furthermore, pro-inflammatory pregnancy conditions such as preeclampsia are associated with an increased risk of ovarian cancer [43] and EVs produced in normal pregnancy induce an anti-inflammatory response in the maternal immune system [44]. In recent reports, placental EVs from pregnancy have been linked to miRNAs containing biological pathways associated with cancer to create a microenvironment that supports immunological privilege and angiogenesis, mediated through cellular reprogramming mechanisms [45,46]. The identified miRNAs in EVs from the maternal circulation prevent tumor metastasis and immunosuppression, inhibit ovarian or endometrial cancer cell proliferation and migration, and promote cancer cell death [5,47].

Another issue is advanced maternal age, parity and medical history of gestational diabetes and chronic hypertension, which are well-known risk factors for preeclampsia and gestational diabetes [48,49]. In the present study, slightly higher maternal age (>35 years), higher proportion of nulliparity, and unestimated CCI were observed in more women with a history of EC and OC than in women in the comparison group. Therefore, the nonsignificant risks of EC and OC in women with a history of preeclampsia and GDM are likely attributable to the specific clinical characteristics of the study population. It is worth noting that the adjusted analysis of these risk factors indicated that an increase in the risk of EC and OC was not observed among women with a history of preeclampsia and GDM, which implies that a history of preeclampsia and gestational diabetes might not influence the risk of EC and OC.

The strength of this study is the large nationally representative number of beneficiaries from Taiwan’s NHIRD, thus minimizing the selection bias. Second, the potential self-report recall bias inherent to the NHIRD can be reduced. This study has some limitations. First, the NHIRD lacks information on parity, multiple GDM pregnancies, cancer histology, subtype and stage, which may affect the accuracy of the outcome. Second, the follow-up period may not be long enough to detect cancer in younger women because of the inherent nature of claims data, which may underestimate cancer risk in younger age groups.

## 10. Conclusions

In the current study, women with a history of GDM were at increased risk of developing BC. There is no convincing evidence that women with a history of EC and OC are at an increased risk for other complications, including preeclampsia, IUGR or LGA, compared with women without a history of EC and OC. Therefore, future studies should determine whether this association is a cause and effect relationship. Using cancer screening to prevent GDM may be an important strategy for inhibiting the development of certain types of cancer. Furthermore, our study highlights the need for further research on tailored cancer surveillance strategies for ethnically diverse GDM patients.

## Figures and Tables

**Table 1:** The baseline characteristics.

Variables	Controls (n = 6714)		EC (n = 328)		OC (n = 502)		BC (n = 5884)		*p*-Value
	n	%	n	%	n	%	n	%	
Complication									
No	5331	79.40	253	77.13	406	80.88	4578	77.80	0.0827
Yes	1383	20.60	75	22.87	96	19.12	1306	22.20	0.0827
Complication									
GDM, gestational diabetes	1253	18.66	71	21.65	86	17.13	1214	20.63	0.0146
Intrauterine growth restriction or slow intrauterine growth	12	0.18	0	0	0	0	11	0.19	0.9649
Large for gestational age newborn	17	0.25	0	0	0	0	14	0.24	0.5562
Preeclampsia	162	2.41	9	2.74	17	3.39	130	2.21	0.3689
Age									<0.001
20–30	298	4.44	23	7.01	63	12.55	170	2.89	
31–40	3697	55.06	182	55.49	305	60.76	3090	52.52	
41–50	2653	39.51	118	35.98	130	25.90	2578	43.81	
>50	66	0.98	5	1.52	4	0.80	46	0.78	
mean, (SD)	39.22	5.10	38.92	5.61	36.92	5.63	39.74	4.85	<0.001
Age at first birth (years, median/range)	35 (32–38)		34 (31–37)		33 (30–36)		34 (32–37)		<0.001
Age at diagnosis (years, median/range)	40 (36–43)		39 (36–42)		37 (33–41)		40 (36–43)		<0.001
Comorbidites									
Myocardial infarction	0	0	0	0	0	0	≤3		0.8713
Congestive heart failure	8	0.12	0	0	0	0	5	0.08	0.7444
Peripheral vascular disease	27	0.40	0	0	≤3		30	0.51	0.7594
Hypertension	354	5.27	21	6.40	25	4.98	299	5.08	0.7414
Cerebrovascular disease	48	0.71	≤3		6	1.20	40	0.68	0.5960
Chronic pulmonary disease	1028	15.31	47	14.33	66	13.15	899	15.28	0.5877
Connective tissue disease	37	0.55	6	1.83	≤3		32	0.54	0.0234
Ulcer disease	1088	16.20	63	19.21	74	14.74	949	16.13	0.3924
Mild liver disease	99	1.47	9	2.74	5	1.00	86	1.46	0.2206
Dyslipidemia	405	6.03	28	8.54	22	4.38	354	6.02	0.1093
Ovarian dysfunction	89	1.33	14	4.27	14	2.79	57	0.97	<0.001
Infertility	989	14.73	78	23.78	93	18.53	825	14.02	<0.001
Obesity	82	1.22	9	2.74	9	1.79	66	1.12	0.0455
Alcohol-related disease	18	0.27	0	0	0	0	22	0.37	0.4927
Charlson comorbidity index									0.1071
1	6602	98.33	≤3		≤3		5789	98.39	
2–3	103	1.53					86	1.46	
>3	9	0.13					9	0.15	
Parity (number, %)									<0.001
1	4766	70.99	273	83.23	363	72.31	4212	71.58	
≥2	1948	29.01	55	16.77	139	27.69	1672	28.42	
Abortion (number, %)									0.022
0	5496	81.86	254	77.44	410	81.67	4737	80.51	
1	919	13.69	49	14.94	70	13.94	874	14.85	
2	217	3.23	13	3.96	16	3.19	198	3.37	
≥3	82	1.22	12	3.66	6	1.20	75	1.27	

Remarks: EC, endometrial cancer; OC, ovarian cancer; BC, breast cancer.

**Table 2:** Multivariate logistic regression analysis of pregnancy complications and history of Gynecological cancers.

Variable	Non-EC (n = 328)		EC (n = 328)		cOR	(95% CI)	*p*-Value	aOR	(95% CI)	*p*-Value
	n	%	n	%						
GDM	65	19.82	71	21.65	1.14	(0.87, 1.49)	0.3306	1.10	(0.84, 1.44)	0.4867
Intrauterine growth restriction or slow intrauterine growth	0		0		0.00					
Large for gestational age newborn	0		0		0.00					
Preeclampsia	10	3.05	9	2.74	0.90	(0.36, 2.24)	0.816	0.91	(0.34, 2.45)	0.849
Variable	Non-OC (n = 502)		OC (n = 502)		cOR	(95% CI)	*p*-value	aOR	(95% CI)	*p*-value
	n	%	n	%						
GDM	85	16.93	86	17.13	1.01	(0.73, 1.41)	0.933	1.02	(0.73, 1.43)	0.896
Intrauterine growth restriction or slow intrauterine growth	0		0		199,762	(0.00, NA)	0.978	501,883	(0.00, NA)	0.986
Large for gestational age newborn					-	-	-	-	-	-
Preeclampsia	11	2.19	17	3.39	1.56	(0.73, 3.37)	0.254	1.87	(0.82, 4.27)	0.139
Variable	Non-BC (n = 5884)		BC (n = 5884)		cOR	(95% CI)	*p*-value	aOR	(95% CI)	*p*-value
	n	%	n	%						
GDM	1103	18.75	1214	20.63	1.13	(1.03, 1.23) *	0.010 *	1.12	(1.02,1.23) *	0.013
Intrauterine growth restriction or slow intrauterine growth	8	0.14	11	0.19	1.37	(0.55, 3.41)	0.496	1.42	(0.57, 3.55)	0.454
Large for gestational age newborn	16	0.27	14	0.24	0.87	(0.43, 1.79)	0.715	0.88	(0.43, 1.80)	0.720
Preeclampsia	141	2.40	130	2.21	0.92	(0.72, 1.17)	0.499	0.92	(0.72, 1.18)	0.519

cOR: crude odds ratio; aOR: adjusted odds ratio; *: *p*-value < 0.05. Comorbidities include myocardial infarction, congestive heart failure, peripheral vascular disease, hypertension, cerebrovascular disease, dementia, chronic pulmonary disease, connective tissue disease, ulcer disease, mild liver disease, dyslipidemia, ovarian dysfunction, infertility, obesity, and alcohol-related disease.

## Data Availability

Data are provided within the manuscript or Supplementary Information file.

## Supplementary Materials

The following supporting information can be downloaded at: https://www.mdpi.com/article/10.3390/medicina61010001/s1, Table S1: International Classification of Disease (ICD) codes.

## References

[1] American Cancer Society. https://www.cancer.org/cancer/ovarian-cancer/about/key-statistics.html.

[2] Health Promotion Administration, Ministry of Health and Welfare. http://www.hpa.gov.tw/bhpnet/English/Index.aspx.

[3] Kaaks R., Lukanova A., Kurzer M.S. (2002). Obesity, endogenous hormones, and endometrial cancer risk: A synthetic review. Cancer Epidemiol. Biomark. Prev..

[4] Setiawan V.W., Pike M.C., Karageorgi S., Deming S.L., Anderson K., Bernstein L., Brinton L.A., Cai H., Cerhan J.R., Cozen W. (2012). Age at last birth in relation to risk of endometrial cancer: Pooled analysis in the epidemiology of endometrial cancer consortium. Am. J. Epidemiol..

[5] Main C., Chen X., Zhao M., Chamley L.W., Chen Q. (2022). Understanding How Pregnancy Protects Against Ovarian and Endometrial Cancer Development: Fetal Antigens May Be Involved. Endocrinology.

[6] Högnäs E., Kauppila A., Hinkula M., Tapanainen J.S., Pukkala E. (2016). Incidence of cancer among grand multiparous women in Finland with special focus on non-gynecological cancers: A population-based cohort study. Acta Oncol..

[7] National Cancer Institute. https://www.cancer.gov/about-cancer/causes-prevention/risk/hormones/reproductive-history-fact-sheet.

[8] Opdahl S., Romundstad P.R., Alsaker M.D., Vatten L.J. (2012). Hypertensive diseases in pregnancy and breast cancer risk. Br. J. Cancer.

[9] Ruiz R., Herrero C., Strasser-Weippl K., Touya D., Louis J.S., Bukowski A., Goss P.E. (2017). Epidemiology and pathophysiology of pregnancy-associated breast cancer: A review. Breast.

[10] Xie F., Liu L., Yang H., Liu M., Wang S., Guo J., Yu L., Zhou F., Wang F., Xiang Y. (2022). The Impact of Reproductive Factors on the Risk of Breast Cancer by ER/PR and HER2: A Multicenter Case-Control Study in Northern and Eastern China. Oncologist.

[11] De Oliveira Andrade F., Verma V., Hilakivi-Clarke L. (2022). Maternal obesity and resistance to breast cancer treatments among offspring: Link to gut dysbiosis. Cancer Rep..

[12] Calderon-Margalit R., Friedlander Y., Yanetz R., Deutsch L., Perrin M.C., Kleinhaus K., Tiram E., Harlap S., Paltiel O. (2009). Preeclampsia and subsequent risk of cancer: Update from the Jerusalem Perinatal Study. Am. J. Obs. Gynecol..

[13] Wang F., Zhang W., Cheng W., Huo N., Zhang S. (2021). Preeclampsia and cancer risk in women in later life: A systematic review and meta-analysis of cohort studies. Menopause.

[14] Jordao H., Herink K., Ka E., McVicker L., Kearns C., McMenamin Ú.C. (2023). Pre-eclampsia during pregnancy and risk of endometrial cancer: A systematic review and meta-analysis. BMC Womens Health.

[15] Liu Y., Chen X., Sheng J., Sun X., Chen G.Q., Zhao M., Chen Q. (2021). Complications of Pregnancy and the Risk of Developing Endometrial or Ovarian Cancer: A Case-Control Study. Front. Endocrinol..

[16] Gill G., Giannakeas V., Read S., Lega I.C., Shah B.R., Lipscombe L.L. (2024). Risk of Breast Cancer After Diabetes in Pregnancy: A Population-based Cohort Study. Can. J. Diabetes.

[17] Fuchs O., Sheiner E., Meirovitz M., Davidson E., Sergienko R., Kessous R. (2017). The association between a history of gestational diabetes mellitus and future risk for female malignancies. Arch. Gynecol. Obs..

[18] Pace R., Rahme E., Dasgupta K. (2020). Gestational diabetes mellitus and risk of incident primary cancer: A population-based retrospective cohort study. J. Diabetes.

[19] Shim S.H., Noh E., Lee A.J., Jang E.B., Kim M., Hwang H.S., Cho G.J. (2023). Risk of adverse obstetric outcomes in patients with a history of endometrial cancer: A nationwide population-based cohort study. BJOG.

[20] Chen Q., Guo F., Jin H.Y., Lau S., Stone P., Chamley L. (2012). Phagocytosis of apoptotic trophoblastic debris protects endothelial cells against activation. Placenta.

[21] Jordan S.J., Na R., Johnatty S.E., Wise L.A., Adami H.O., Brinton L.A., Chen C., Cook L.S., Dal Maso L., De Vivo I. (2017). Breastfeeding and Endometrial Cancer Risk: An Analysis From the Epidemiology of Endometrial Cancer Consortium. Obs. Gynecol..

[22] Roy D., Liehr J.G. (1999). Estrogen, DNA damage and mutations. Mutat. Res..

[23] Chen G.G., Zeng Q., Tse G.M. (2008). Estrogen and its receptors in cancer. Med. Res. Rev..

[24] Kim J.J., Chapman-Davis E. (2010). Role of progesterone in endometrial cancer. Semin. Reprod. Med..

[25] Henderson B.E., Feigelson H.S. (2000). Hormonal carcinogenesis. Carcinogenesis.

[26] Mauro L.J., Spartz A., Austin J.R., Lange C.A. (2023). Reevaluating the Role of Progesterone in Ovarian Cancer: Is Progesterone Always Protective?. Endocr. Rev..

[27] Wan J., Gao Y., Zeng K., Yin Y., Zhao M., Wei J., Chen Q. (2016). The levels of the sex hormones are not different between type 1 and type 2 endometrial cancer. Sci. Rep..

[28] Placenta: Overview, Anatomy, Function & Complications. https://my.clevelandclinic.org.

[29] Russo J., Moral R., Balogh G.A., Mailo D., Russo I.H. (2005). The protective role of pregnancy in breast cancer. Breast Cancer Res..

[30] Nechuta S., Paneth N., Velie E.M. (2010). Pregnancy characteristics and maternal breast cancer risk: A review of the epidemiologic literature. Cancer Causes Control.

[31] Berkane N., Liere P., Lefevre G., Alfaidy N., Abi Nahed R., Vincent J., Oudinet J.P., Pianos A., Cambourg A., Rozenberg P. (2018). Abnormal steroidogenesis and aromatase activity in preeclampsia. Placenta.

[32] Baud O., Berkane N. (2019). Hormonal Changes Associated With Intra-Uterine Growth Restriction: Impact on the Developing Brain and Future Neurodevelopment. Front. Endocrinol..

[33] Trabert B., Troisi R., Grotmol T., Ekbom A., Engeland A., Gissler M., Glimelius I., Madanat-Harjuoja L., Sørensen H.T., Tretli S. (2020). Associations of pregnancy-related factors and birth characteristics with risk of endometrial cancer: A Nordic population-based case–control study. Int. J. Cancer.

[34] Ebrahim Valojerdi A., Janani L. (2018). A brief guide to propensity score analysis. Med. J. Islam. Repub. Iran..

[35] Parsons L.S., Ovation Research Group (2001). Reducing Bias in a Propensity Score Matched-Pair Sample Using Greedy Matching Techniques.

[36] Taiwan’s Declining Birth Rate Difficult to Reverse: Official. https://focustaiwan.tw/society/202410240013.

[37] Peng Y.S., Lin J.R., Cheng B.H., Ho C., Lin Y.H., Shen C.H., Tsai M.H. (2019). Incidence and relative risk for developing cancers in women with gestational diabetes mellitus: A nationwide cohort study in Taiwan. BMJ Open.

[38] Slouha E., Gates K.M., Al-Geizi H., Baah E., Clunes L.A., Kollias T.F. (2024). The Relationship Between Gestational Diabetes and the Risk of Cancer: A Systematic Review. Cureus.

[39] Tan V.M.H., Lee Y.S., Venkataraman K., Khoo E.Y.H., Tai E.S., Chong Y.S., Gluckman P., Leow M.K.S., Khoo C.M. (2015). Ethnic differences in insulin sensitivity and beta-cell function among Asian men. Nutr. Diabetes.

[40] Ma R.C., Chan J.C. (2013). Type 2 diabetes in East Asians: Similarities and differences with populations in Europe and the United States. Ann. N. Y. Acad. Sci..

[41] Gao H., Salim A., Lee J., Tai E.S., Van Dam R.M. (2012). Can body fat distribution, adiponectin levels and inflammation explain differences in insulin resistance between ethnic Chinese, Malays and Asian Indians?. Int. J. Obes..

[42] Seah J.Y.H., Sim X., Khoo C.M., Tai E.S., van Dam R.M. (2023). Differences in type 2 diabetes risk between East, South, and Southeast Asians living in Singapore: The multi-ethnic cohort. BMJ Open Diabetes Res. Care.

[43] Lorincz A.M., Sukumar S. (2006). Molecular links between obesity and breast cancer. Endocr. Relat. Cancer.

[44] Katsanis W.A., Shields L.B., Spinnato J.A., Gerçel-Taylor C., Taylor D.D. (1998). Immune recognition of endometrial tumor antigens induced by multiparity. Gynecol. Oncol..

[45] Shields L.B., Gerçel-Taylor Ç., Yashar C.M., Wan T.C., Katsanis W.A., Spinnato J.A., Taylor D.D. (1997). Induction of immune responses to ovarian tumor antigens by multiparity. J. Soc. Gynecol. Investig..

[46] Tiozzo C., Bustoros M., Lin X., De Mejia C.M., Gurzenda E., Chavez M., Hanna I., Aguiari P., Perin L., Hanna N. (2021). Placental extracellular vesicles-associated microRNA-519c mediates endotoxin adaptation in pregnancy. Am. J. Obs. Gynecol..

[47] Pillay P., Vatish M., Duarte R., Moodley J., Mackraj I. (2019). Exosomal microRNA profiling in early and late onset preeclamptic pregnant women reflects pathophysiology. Int. J. Nanomed..

[48] Bakkali M.E., Derdaki M., Quyou A. (2024). Advanced Maternal Age, Gestational Diabetes, and Parity: A Moderated Mediation Model for Preeclampsia. Tanzan. J. Health Res..

[49] Yang Y., Wu N. (2022). Gestational Diabetes Mellitus and Preeclampsia: Correlation and Influencing Factors. Front. Cardiovasc. Med..

