# Supplementary material for: Association of Pregnancy Complications with Endometrial or Ovarian or Breast Cancer: A Case Control Study
Source: Medicina (Kaunas). 2024 Dec 24;61(1):1. doi: 10.3390/medicina61010001 (PMC11766899; doi:10.3390/medicina61010001)
Supplement: Supplementary file 1 [file medicina-61-00001-s001.zip › medicina-3324816-supplementary.pdf]

Supplementary Material pregnancy complications

Supplementary Table S1. International Classification of Disease (ICD) codes

NORMAL DELIVERY, AND OTHER INDICATIONS FOR CARE IN PREGNANCY, LABOR, AND DELIVERY ICD-9 Code range 650-659.
Endometrial cancer, EC : ICD-10-CM: C54.1, C55, D07.0, N85.02
ICD-9: 179; 181, 182-182.1, 182.8 ;233.2, 236.0, 236.1, 621.33
Ovarian cancer, OC: ICD-9: 183.0-183.9; 236.2; ICD-10: C56, C56.1, C56.2, C56.9
Other unspecified female genital organs:184.0-9; 233.3, 233.30-233.32; 233.39; 236.3<br>ICD-10: C51-C58
Breast cancer:ICD-10: C50.011-012, C50.111-112, C50.611-612, C50.811-812, C50.911-912, D05.01-D05.02, D05.11-12, D05.81-82
GDM (Gestation diabetes mellitus): ICD-9: 648.8, 648.83; ICD-10: O24. 419
T2DM: ICD-9:250, ICD-9:250-1, 250-3; ICD-10:E11
Charlson Comorbidity Index : Co-morbidities(risk factors)
1 score<br>Myocardial infarction<br>ICD-9: 410; ICD-10: I21, I22, I23<br>Congestive heart failure<br>ICD-9: 427.09, 427.10, 427.11, 427.19, 428.99, 782.49; ICD-10: I50, I11.0, I13.0, I13.2<br>Peripheral vascular disease<br>ICD-9: 440, 441, 442, 443, 444, 445; ICD-10: I70, I71, I72, I73, I74, I77<br>Hypertension:<br>ICD-9: 401.9; ICD-10: I10<br>Cerebrovascular disease<br>ICD-9: 430-438; ICD-10: I60-I69, G45, G46<br>Dementia<br>ICD-9: 290.09-290.19, 293.09; ICD-10: F00-F03, F05.1, G30<br>Chronic pulmonary disease<br>ICD-9: 490-493, 515-518, ICD-10: J40-J47, J60-J67, J68.4, J70.1, J70.3, J84.1, J92.0, J96.1, J98.2, J98.3<br>Connective tissue disease<br>ICD-9: 712, 716, 734, 446, 135.99; ICD-10: M05, M06, M08, M09, M30, M31, M32, M33, M34, M35, M36, D86<br>Ulcer disease<br>ICD-9: 530.91, 530.98, 531-534; ICD-10: K22.1, K25-K28<br>Mild liver disease<br>ICD-9: 571, 573.01, 573.04; ICD-10: B18, K70.0-K70.3, K70.9, K71, K73, K74, K76.0<br>Diabetes types 1 and 2<br>ICD-9: 249.00, 249.06, 249.07, 249.09, 250.00, 250.06, 250.07, 250.09; ICD-10: E10.0, E10.1, E10.9, E11.0, E11.1, E11.9

**Dyslipidemia:**<br>**Ovarian dysfunction:ICD-10: E 28.0-28.9**<br>**Infertility: ICD-10: N46.8-46.9 ; ICD-10: N97.0-97.9**
**2 score**<br>**Hemiplegia**<br>**ICD-9: 344; ICD-10: G81, G82**<br>**Moderate to severe renal disease**<br>**ICD-9: 403, 404, 580-583, 584, 590.09, 593.19, 753.10-753.19, 792; ICD-10: 12, I13, N00-N05, N07, N11, N14, N17-N19, Q61**<br>**Diabetes with end-organ damage**<br>**ICD-9: 249.01-249.05, 249.08, 250.01-250.05, 250.08; ICD-10: E10.2-E10.8, E11.2-E11.8**<br>**Any tumor ICD-9: 140-194; ICD-10: C75-C75.9**<br>**Leukemia**<br>**ICD-9: 204-207; ICD-10: C91-C95**<br>**Lymphoma**<br>**ICD-9: 200-203, 275.59; ICD-10: C81-C85, C88, C90, C96**<br>**Breast cancer: ICD-9: 170, ICD-10: C50**<br>**Prostate cancer: ICD-9: 185 , 233.4 , 222.2; ICD-10-CM: C61**<br>**Bladder cancer: ICD-9: 188.9; ICD-10-CM C67.9**<br>**Lung/ bronchus: ICD-9 :162.9**<br>**Colon: ICD-9-CM 153.9; ICD-10-CM C18.9**<br>**Rectal: ICD-9-CM 154.0, 154.1 and 154.8; ICD-10 C20**<br>**Pancreas: ICD-9-CM 157.0-157.9**<br>**Kidney/Renal: ICD-9-CM 189.0; ICD-10-CM C64.9**
**Diabetes with end-organ damage**<br>**ICD-9: 249.01-249.05, 249.08, 250.01-250.05, 250.08; ICD-10: E10.2-E10.8, E11.2-E11.8**
**3 score**
**Moderate to severe liver disease**<br>**ICD-9: 070.00, 070.02, 070.04, 070.06, 070.08, 573.00, 456.00-456.09;**<br>**ICD-10: B15.0, B16.0, B16.2, B19.0, K70.4, K72, K76.6, I85**
**6 score**
**Metastatic solid tumor**<br>**ICD-8: 195-198, 199; ICD-10: C76-C80**<br>**AIDS**<br>**ICD-8: 079.83; ICD-10: B21-B24**
**Obesity: ICD-9: 277.99. ICD-10: E66.0-66.9**
**Alcohol-related disease**<br>**ICD-9: 291.00–291.99,303.00–303.99, 571.09, 571.10, 577.10, E861, N979, N980; ICD-10: E24.4, E52.9A, F10.0, F10.1, F10.2–10.9, G31.2, G62.1,G72.1, I42.6, K29.2, K70, K85.2, K86.0, T50.0A, T51, R78, Z50.2, Z71.4, Z72.1.**
